# Hyperæmia and Inflammation of the Dental Pulp

**Published:** 1897-08

**Authors:** P. P. Nelson


### Hyperæmia and Inflammation of the Dental Pulp.

BY P. P. NELSON, D.D.S.

It is generally considered that the physical function of the pulp is the formation of the dentine and the maintenance of its vitality. But aside from this the pulp has a special sensory function which is limited and has no parallel among the organs and tissues of the body.

This function consists in a peculiar resentment to thermal changes.

In diseases of organs having a special function, the expressions of disease are exaggerated during the exercise of that function. With many organs of the body, the performance of their special function is necessary to the continued existence of the individual, but in all cases when rest can be had, it seems to be a plain duty to secure it. The oculist shields from the light, the inflamed retina or iris, in order that the inflamed tissue may have rest from the performance of its special function. On the same principle, should not a diseased dental pulp be carefully protected from thermal changes? May it not be, that a large proportion of the difficulty that arises in the pulps of the teeth under treatment, is due to carelessness in this particular?

The most important affection, perhaps, because it is the most common, that the dentist has to combat, and because it so often terminates in the death of the organ, is hyperæmia of the dental pulp? Dr. Black says, that hyperæmia may occur in any degree, from a slight distension of the vessels to an expansion that seems enormous, caused by excessive thermal changes.

Sensitiveness to thermal changes, in a certain degree, is the normal sensory function of the pulp. In each instance of the exercise of this function, there is an unusual amount of blood sent to the pulp. This, when in a reasonable degree, is a temporary physiological hyperæmia which calls out a simple warning in the form of an unpleasant sensation, and immediately passes away. In this case no injury results; but let this be repeated frequently, with an unusual degree of thermal change, and the vessels will finally fail to contract in their normal manner, and remain overfilled with blood and also acquire an unusual degree of susceptibility to thermal influences, so that very slight changes produce great results. Primary among the causes which produce this result may be mentioned, the careless use of the burr, over-heating it, in the preparation of the cavity and the introduction of a metal-filling in close proximity to the pulp, without an intervening non-conductor.

The symptoms of hyperæmia is pain, sharp and lancinating and paroxysmal, especially in its early stages. It is usually referred to the teeth, but it is not unusual for the patient to designate the wrong tooth, or be unable to locate the pain in any particular tooth, or it may be referred to any part of the distribution of the fifth nerve, as in some forms of facial neuralgia.

Inflammation of the dental pulp is the next most common affection with which we as dentists have to deal, and which is but one step removed from hyperæmia, in fact hyperæmia is the first step in the inflammatory process, and besides the local plethora of the blood vessels of the pulp, as in hyperæmia, we now have the usual results of an inflammation as found in other portions of the body, going through the same stages in a manner similar to other tissues of the body, with the exception of swelling and pain, being confined in a resistant, bony canal, it has no room to swell, except in cases of exposure. The pain is out of all proportion to the tissue affected and the extent of inflammation.

Among the causes of inflammation of the pulp are either exposure of the pulp by decay, some of the forms of abrasion, mechanical violence in the form of accident, or the operations of the dentist in the preparation of cavities and the insertion of fillings.

